# 12-Year Case Series of Patients with Heat Illness from an Urban Hospital System in the American Southwest

**DOI:** 10.5811/westjem.49002

**Published:** 2026-02-03

**Authors:** Megan McElhinny, Logan Garr, Tristan Chen, Brandon Garcia, Bikash Bhattarai, Liliya Kraynov, Geoff Comp

**Affiliations:** * Creighton University School of Medicine-Phoenix, Department of Emergency Medicine, Phoenix, Arizona; † University of Arizona, College of Medicine-Phoenix, Department of Emergency Medicine, Phoenix, Arizona; ‡ Valleywise Medical Center, Department of Emergency Medicine, Phoenix, Arizona

## Abstract

**Objectives:**

Climate change has led to more frequent and intense heat events with dramatic increases in heat illness and heat-related deaths. We compared demographic characteristics such as age, sheltering status, and underlying health conditions that contribute to susceptibility to extreme heat. We described the clinical course of these patients, presenting over a 12-year span, who were diagnosed with heat-related illness, to inform local risk stratification.

**Methods:**

We conducted retrospective chart abstraction of encounters between January 1, 2012–December 31, 2023, which included adults 18–89 years of age, presenting to a single hospital system’s emergency department (ED), with an International Classification of Diseases, 10th Revision, discharge diagnosis within the T67 heat-related diagnosis code family. We compared demographic characteristics to baseline ED presentations and summarized clinical characteristics in frequencies. Trends were described over time juxtaposed with temperature data.

**Results:**

The 141 patients with a heat illness diagnosis were older, with a mean age of 53, and were more likely to be male (81.6%), White (51.8%), or Native American (7.8%) as compared to adult (18–89 years of age) all-comer ED presentations. Patients with a heat illness often carried co-occurring diagnoses of contact burns (38.3%) or rhabdomyolysis (25.5%). Common chronic comorbid conditions included cardiovascular disease (33.3%) and substance use disorder (22.0%). Antipsychotics (22.0%), laxatives (24.1%), and beta blockers (15.6%) were frequent home medications among heat-affected patients. Of the patients who were the most critically ill from heat illness, 35.5% required ED intubation and 95.7% were admitted, with 45.9% of those requiring intensive care. While most were discharged to self-care (59.3%), 26.7% required skilled nursing care at discharge.

**Conclusion:**

This review describes the characteristics and clinical course of patients diagnosed with heat illness over more than a decade of increasingly frequent and extreme heat in Phoenix, AZ. It provides a unique and sizeable cohort that can guide the surveillance and treatment of heat illness. We highlight clinical trends and gaps in clinical heat illness data to identify vulnerabilities and protective factors among our patients.

## INTRODUCTION

### Background

Climate change has led to a surge in extreme heat events, with rising temperatures posing significant threats to public health.^1^ Phoenix, Arizona, stands at the forefront of this climatic challenge, experiencing an increasing frequency and intensity of extreme heat events.^2^ The Maricopa County Public Health Department recorded 645 heat-related deaths in 2023, nearly double the deaths recorded just two years prior.^3^ In 2023 Phoenix experienced 133 days with a temperature greater than 100 ˚F and 55 days greater than 110 ˚F.^4^ Seasonal highs are increasingly more extreme and prolonged, particularly in the southwest United States (US). California, Arizona, and Texas, with similar hot climates, represent only 23% of the US population but accounted for a disproportionate 37% of heat deaths between 2004–2018.^5^

Of US climate-related fatality rates in 2023, extreme heat events were the deadliest.^6^ Factors such as demographics and underlying health conditions may contribute to an increased susceptibility to the adverse effects of extreme heat.^7^ In 2023, unsheltered people experienced an estimated 500-fold increased risk of death from a heat-related cause,^8^ representing 45% of heat-related deaths.^3^ In Maricopa County 75% of heat-related deaths occurred outdoors in 2023, and unsheltered people using alcohol or illicit substances experience disproportionate consequences of extreme heat.^9^ Of the 419 deaths in 2023, 65% were associated with substance use, and 62% occurred among those experiencing homelessness.^3^

### Importance

The implications of extreme heat on healthcare are far-reaching.^3^ The increasing incidence of heat-related disease imposes a growing and resource-intensive demand on clinicians and healthcare systems. Emergency medicine stands at the forefront of recognizing, resuscitating, and contributing to risk reduction of populations especially vulnerable to heat illness. An examination of patient characteristics and clinical course is vital to inform focused interventions and improve risk stratification.

### Goals of This Investigation

In this retrospective chart review we compiled a sizeable case series to describe the multifaceted nature of heat illnesses in Phoenix. Our aim in this study was to better understand how the demographics of this cohort vary from the general ED population, to observe the frequency of documented heat-illness risk factors and to identfiy the resources required to care for these patients, informing clinical heat idenfication and treatment strategies.

## METHODS

### Study Design

In this observational study we used a retrospective chart review to describe and analyze patient demographics, patient characteristics, and clinical course among those diagnosed with a heat-related illness. Programmed data extraction by our electronic health record (Epic Systems Incorporated, Verona, WI) data analysts was used to identify cases over the study period and to compile an a priori list of patient characteristic variables for each encounter. These data were cleaned by ordering and grouping by unique clinical identifier. Duplicate records were removed. We manually sorted home medications based on a priori categories of high-risk medications (Appendix 3).^7^ Comparison ED all-comer data among adults (18–89 years of age) over the same study period were collected using Epic Slicer Dicer, a reporting and data visualization tool.

**Population Health Research Capsule**

What do we already know about this issue?

*Among US climate-related fatalities, extreme heat events were the deadliest in 2023, including 419 heat-related deaths in Maricopa County, Arizona*.

What was the research question?

*We described the clinical course of patients diagnosed with heat-related illness over 12 years at a safety-net hospital system*.

What was the major finding of the study?

*Patients were predominantly White (52%) males (82%) with a mean age of 53; 96% were admitted, and 27% were discharged to skilled nursing*.

How does this improve population health?

*These new clinical data, among a sizable cohort, highlight fundamental gaps in identifying and collecting data in affected populations and inform surveillance and risk stratification*.

Retrospective review included programmed extraction by data analysts for clinical characteristics and a two-reviewer manual chart review for clinical course outcomes. Outcomes data were collected by two trained reviewers (LG, BG) who reviewed encounters independently using a standard data collection form. Reviewers were trained using cohorts of 5–10 patients with review and comparison of variables in Research Electronic Data Capture, hosted at Valleywise Health, for consistency and agreement. Variations in abstraction were adjudicated by consensus among the reviewers and a senior author (MM). We modeled chart review methods using Strengthening the Reporting of Observational Studies in Epidemiology guidelines, including clear eligibility criteria, an a priori data-collection form, and trained reviewers with regular reassessment consistent with best practices in emergency medicine observational research and chart review.^8^–^10^ This study was reviewed by the local institutional review board (IRB) and deemed exempt.

### Setting and Selection of Participants

We reviewed patient records from an urban, safety-net hospital system that included two adult emergency departments (ED) and a burn center ED in Phoenix, AZ. Adults 18–89 year of age with an ED visit between January 1, 2012–December 31, 2023, were included. We included patients if they had any ED disposition diagnosis of heat-related illness from the *International Classification of Diseases 10^th^ Revision* (*ICD-10*) diagnosis class T67 family (Appendix A). Pediatric patients were excluded from this study. The IRB stipulated that patients >89 years of age be excluded as they were identifiable within our health system.

### Outcomes

Our primary objective was to describe the characteristics and clinical course of patients diagnosed with heat illness in the ED. Variable selection was based on literature citing risk factors for heat illness, including comorbid conditions, medications, and social demographics.^7^,^13^ A priori demographic characteristics included age, sex, race, ethnicity, ZIP code, and payor source, with employment status and housing status inadequately captured. Clinical characteristics extracted included comorbidities previously identified as risk factors (based on available diagnostic groupers in Appendix B), commonly co-occurring clinical diagnoses, and home medications known to predispose patients to heat illness.^7^,^11^ We also assessed return ED visits within 30, 60, and 90 days from the index visit. Manual, two-reviewer chart review assessed outcomes that were difficult to obtain with programmed extraction alone. These variables included ED length of stay (LOS), whether ED intubation was performed, ED disposition, admission level of care, admission LOS, admission disposition, and gross neurological status on discharge. Secondary outcomes included publicly available climate data from the National Weather Service, including maximum and minimum temperatures in the Phoenix area over the study period.^14^

### Analysis

We summarized demographics, clinical characteristics and clinical course using descriptive data, including frequencies and percentages. The number of patient visits with an *ICD-10* heat-related diagnosis per year was represented visually over time, juxtaposed against daily temperature highs and lows.^14^ Given the nature of this descriptive analysis among a case series, no causal inference or other hypothesis testing analysis was appropriate.

## RESULTS

### Patient Demographic Characteristics

Patients with an ED discharge diagnosis related to heat illness were older, with a mean age of 53 years (23–84), compared to a mean age of 41 (18–89) among all ED presentations for adults aged 18–89. Among the studied patients, 115 (81.6%) were male compared to 50.1% (n=349,829) in the comparison population.

White (51.8%, n = 73) and Native American (7.8%, n = 11) patients were represented at a greater rate in our study population as compared to the all-comer adult population, 20.2% (n = 141,176) and 3.6% (n = 25,293), respectively. In contrast, fewer Blacks were represented in our study population at 10.6% (n = 15) compared to 15.3% (n = 106,894). Hispanic or Latino ethnic groups were represented at a lower rate at 25.5% (n = 36) compared to 48.9% (n = 341,575). More common in our study sample were patients with a documented primary payor source of Medicaid at 57.4% (n = 81) vs 45.8% (n = 319,540), or commercial insurance at 16.3% (n = 23) vs 1.3% (n = 9,011). Fewer patients without insurance at 7.8% (n = 11) vs 27.8% (n = 193,999) were represented in our study population.

Patients’ occupations and housing status were not reliably documented in either population. Documented housing status and occupation were absent in all charts electronically reviewed. Of the study population, 34.8% (n = 49) vs 16.3% (n =. 113,854) claimed 85008 as their home ZIP code, which includes our primary medical center. The second and third most common ZIP codes among the study population resided adjacent to our medical center, including 85007 at 7.1% (n = 10) vs 3.2% (n = 22,258) and 85006 at 5.0% (n = 7) vs 3.9% (n = 27,342), respectively. The next most represented ZIP codes were 85040 (5.0% [n = 7] vs 4.9% [n = 34,314]), which is not directly adjacent to the primary medical center, and 85018 (2.8% [n = 4] vs 1.6% [n = 11,322]), which is adjacent to a secondary medical center opened in 2019 (Table 1).

### Patient Clinical Characteristics

A large segment of the study population was given a diagnosis code of contact burns (38.3%, n = 54) or rhabdomyolysis (25.5%, n = 36), but fewer received a diagnosis of altered mental status (5.7%, n = 8). Notably, our main adult ED remains a regional burn referral center. Frequency of comorbidities included cardiovascular disease (33.3%, n = 47), substance use disorder (22.0%, n = 31), respiratory disease (8.5%, n = 12), diabetes (8.5%, n = 13), and obesity (8.5%, n = 12).

Typical blood pressure agents on patient home medication lists included beta blockers (15.6% n = 22) and calcium channel blockers (15.6% n = 22). Laxatives were a common medication among the study population (24.1%, n = 34). While we did not collect information on mental illness among our study population, antipsychotics (22.0%, n = 31) were common, as was lithium (11.3%, n = 16). Additionally, 7.8% (n = 11) of study patients had a benzodiazepine on their home medication list. The broad category of anticholinergics, including everything from breathing treatments (ipratropium) to allergy medication (diphenhydramine), was included in the home medications of 18 study participants (12.8%). Antihistamine medications were also prevalent at 17.0% (n = 24) (Table 2).

This cohort represents a high-acuity group, likely those with the most severe forms of heat illness. Most patients dispositioned from the ED with a diagnosis of heat-related illness were admitted (95.7%, n = 135), with 4.3% (n = 6) discharged to home. No patients were deceased at ED disposition. While in the ED, 35.5% (n = 50) required intubation. Of those who were admitted to the hospital, 45.9% (n = 62) were admitted to the intensive care unit (ICU), 25.9% (n = 35) were admitted to progressive/step-down units and 28.1% (n = 38) to medical/surgical units. The median time to admission was 5 hours 19 minutes (1hour 22 minutes-22 hours 16 minutes) while the median hospital LOS was 2 days 18 hours 42 minutes (9 hours 54 minutes-63 days 18 hours 30 minutes). Of all patients alive at ED or hospital disposition, 94% (n = 125) were grossly neurologically intact at discharge based on the exam detailed at discharge. Patients were most commonly discharged from hospital admission to self-care (59.3%, n = 80) followed by skilled nursing facility (SNF) (26.7%, n = 36), inpatient behavioral health (5.7%, n = 8), and legal custody (2.2%, n = 3). Eight patients (5.9% of admitted patients) died during their inpatient hospitalization (Table 3).

### Climate Trends and Frequency of Heat-related Illness

Most study patients presented within the meteorological summer months of June–August, with a few outliers presenting in the shoulder seasons of May and September. Over the study period, the number of cases per year gradually increased in synchrony with the increasing number of yearly days equal to or above 105 °F. We observed a notable uptick in the number of days equal to or above 105 °F, which corresponded with an increase in total cases per year between 2018–2023.

## LIMITATIONS

This study has several limitations that must be acknowledged to contextualize the findings and guide future research. This study was a retrospective chart abstraction and depended on existing medical records. The quality of clinical data, including the home medication list, was limited by programmed EHR extraction. Reliance on diagnostic codes to identify heat illness underestimates its incidence due to variability in physician coding. We suspect that recency bias also plays a role in the number of cases presenting and identified over time, with increasing average temperatures over the study period and the introduction of a cold-water immersion protocol in 2018. Given the large number of heat-related deaths reported in Maricopa County, and with recent emergency medical services collaboration, we understand that our medical center treats, at most, approximately one-third of the patients being transported in the downtown metro area of Phoenix. In addition, subsequent efforts to identify heat illness cases by other methods or parameters suggest that identification by diagnostic code significantly underestimates the burden of disease at our medical center. Finally, including burn ED presentations and excluding the extremes of age (< 18 and > 89 years of age) under-represents the full spectrum of susceptibility and response to heat illnesses across all age groups and limits generalizability.

## DISCUSSION

This observational study reviews a sizeable cohort of 141 patients with heat-related illness who were most acutely and severely ill over a 12-year period, which represents a unique perspective. Male and non-Hispanic patients comprised a higher proportion of our study population than the general ED population. Specifically, Native American patients were over-represented in our heat-illness cohort. Disparities among subgroups are hypothesis-generating and require further investigation by delineating whether this subgroup successfully employs tactics to avoid heat injury, does not present to the ED when they experience a heat illness, or physicians are less likely to code this population as experiencing a heat illness. Heat injury patterns such as occupational exposure, unsheltered status, concomitant use of mind-altering substances, the use of stimulants that increase metabolic heat, as well as housing vulnerabilities such as the use of prefabricated homes or unstable access to adequate utilities, may contribute, as highlighted in the annual Maricopa County Heat Death reporting.^3^ Similar investigations among local universities, including a white paper in 2024 from the University of Arizona, highlight homelessness, drugs, social isolation, and lack of air conditioning as significant vulnerabilities.^15^ To the extent that risk factors could be reliably measured among this cohort, demographics among those affected by heat illness are comparable to population data published by Maricopa Public Health. There is consistency between this cohort regarding indicators of substance use disorder. Additional cross-sectional detail is available from this cohort on the co-occurrence of the prescription of medications typically used in the treatment of mental health.

The most reported home ZIP codes for patients affected by heat illness represent areas spatially adjacent to our medical centers, with even greater representation of the local ZIP codes than in the comparator population. Further investigation of local climate and demographic data, along with multicenter expansion of the study, would further clarify whether these observations relate to higher incidence or risk vs more frequent presentation or identification bias.

The T67 *ICD-10* diagnosis code family is diverse; this cohort is a high-acuity representation, with most (95.7%) requiring admission, and more than a third (45.9%) of admitted patients requiring intensive care level of service. Despite the acuity, the mortality in this heterogeneous group of 5.9% falls behind cited heat stroke mortality rates between 15–71%, and is more comparable to specific mortality rates for rapidly treated exertional heat stroke.^13^ Heat-related illness is typically described with significant morbidity, and 26.7% of patients in our cohort required skilled nursing care after their hospital discharge. Our cohort also required significant resources while in the ED, spending on average 5 hours and 19 minutes in the ED before being admitted, with 35.5% requiring ED intubation. Our cohort suggests encouraging functional outcomes, with 67.4% surviving their hospitalization and not requiring SNF placement. While our chart review reports 94% were neurologically intact at the time of disposition, more detailed and accurate measures of functional status are likely necessary, as close to one-fourth of patients with heat stroke (34.4%) suffered long-standing neurological deficits in a 2018 Australian case series.^17^

The total number of patients captured by diagnostic code data is likely to be lower than the total number of patients facing heat illness. Given that climate change continues to intensify, clinicians and public health officials need to find more reliable ways to monitor heat illness. We are aware of a limited and growing number of sizeable cohorts described with heat illness, most of which have limited clinical data. This study provides a unique perspective with demographic detail and outcome measures and is more nuanced than prior studies.^18^,^19^ This cohort largely corroborates observational data previously published on heat-illness risk factors.^20^,^21^ While vulnerable populations are more predisposed to heat illness due to multifactorial reasons, further investigation into social risk factors of patients affected by heat illness should be a focus for public health surveillance and population outreach to both patients and clinicians.

## CONCLUSION

As extreme heat events become more frequent and intense, our patients, practitioners, and healthcare systems must anticipate, adapt, and act swiftly. Our cohort, collected over 12 years, including patients presenting to the emergency department and found to have a heat-related illness, provides new clinical details among this sizable cohort. It highlights fundamental gaps in identifying and collecting of data in affected populations. This review provides a new perspective on previously published population data and provides limited clinical detail from the emergency medicine perspective, with the ED often serving as a social safety net.

## Supplementary Information



## Figures and Tables

**Table 1:** Patient demographics in a study of heat illness and heat-related deaths in Phoenix, Arizona.

Total	Heat-related diagnosis (N = 141)	Adult (18–89 years) ED encounters (N = 698,235)
Age (median [range])	53 years (23–84)	41 years (18–89)
Male sex (% ([n])	81.6% (n = 115)	50.1% (n = 349,829)
Race/ethnicity (% [n])		
White	51.8% (n = 73)	20.2% (n = 141,176)
Black	10.6% (n = 15)	15.3% (n = 106,894)
Hispanic	25.5% (n = 36)	48.9% (n = 341,575)
Native American	7.8% (n = 11)	3.6% (n = 25,293)
Asian	0.7% (n = 1)	1.4% (n = 9,671)
Primary payor (%(n)		
Medicaid	57.4% (n = 81)	45.8% (n = 319,540)
Medicare	11.3% (n = 16)	11.2% (n = 78,286)
Commercial Insurance	16.3% (n = 23)	1.3% (n = 9,011)
Uninsured	7.8% (n = 11)	27.8% (n =193,999)
Other	6.4% (n =. 9)	14.0% (n = 97,407)
Patient’s Primary ZIP Code		
85008	34.8% (n = 49)	16.3% (n = 113,854)
85007	7.1% (n = 10)	3.2% (n = 22,258)
85006	5.0% (n = 7)	3.9% (n = 27,342)
85040	5.0% (n = 7)	4.9% (n = 34,314)
85018	2.8% (n = 4)	1.6 (n = 11,322)

*ED*, emergency department.

**Table 2:** Comorbid conditions and medications of patients in a study of of heat illness and heat-related deaths.

Comorbid conditions	
Past medical history	
Cardiovascular	33.3% (n = 47)
Respiratory	8.5% (n = 12)
Diabetes	8.5% (n = 12)
Obesity	8.5% (n = 12)
Substance use	22.0% (n = 31)
Co-occurring diagnoses	
Altered mental status	5.7% (n = 8)
Contact burns	38.3% (n = 54)
Rhabdomyolysis	25.5% (n = 36)
Medications	
Anticholinergic	12.8% (n = 18)
Antihistamine	17.0% (n = 24)
Antipsychotic	22.0% (n = 31)
Benzodiazepines	7.8% (n = 11)
Beta blockers	15.6% (n = 22)
Calcium channel blockers	15.6% (n = 22)
Diuretics	10.6% (n = 15)
Laxatives	24.1% (n = 34)
Lithium	11.3% (n = 16)
SSRI	1.4% (n = 2)
TCA	1.4% (n = 2)
Weight loss medication	0.7% (n = 1)

*SSRI*, selective serotonin reuptake inhibitor; *TCA*, tricyclic antidepressant.

**Table 3:** Clinical course of patients in study of relationship between rising temperatures and heat-related illnesses.

Required ED intubation	35.5% (n = 50)
ED disposition	
Discharged	4.3% (n = 6)
Admitted	95.7% (n = 135)
Deceased	0% (n = 0)
Time to admission if admitted (median, IQR)	5 hours 19 minutes (1hr 22min–22hr 16min)
Hospital admission level of care	95.7% (n =135)
Med/Surg	28.1% (n = 38)
Progressive/Step down	25.9% (n = 35)
ICU	45.9% (n = 62)
Length of hospital admission	
Median (range)	2 days 18hr 42min (9hr 54 min–63d 18hr 30min)
Neuro status if alive on disposition	
Neuro intact	94.0% (n = 125)
Hospital disposition	
Self-care	59.3% (n = 80)
Behavioral health	5.9% (n = 8)
Custody	2.2% (n = 3)
Rehab	0% (n = 0)
SNF	26.7% (n = 36)
Deceased	5.9% (n = 8)
Return ED visits	
0–30 days	6.4 (n = 9)
31–60 days	3.6 (n = 5)
61–90 days	0% (n = 0)

*ED*, emergency department; *ICU*, intensive care unit; *SNF*, skilled nursing facility; *IQR*, interquartile range.
