# Supplementary material for: 12-Year Case Series of Patients with Heat Illness from an Urban Hospital System in the American Southwest
Source: West J Emerg Med. 2026 Feb 3;27(2):291–7. doi: 10.5811/westjem.49002 (PMC13016062; doi:10.5811/westjem.49002)
Supplement: Supplementary file 1 [file wjem-27-291-s001.pdf]

Appendix A:

List of Diagnosis codes:[22]

Heat exhaustion T67.5

Collapse R55- heat T67.1

Cramps R25.2- heat T67.2

Heat exhaustion or prostration T67.4

Heat, exhaustion T67.5

Heat fatigue (transient) T67.6

Heat (effect)

- apoplexy T67.9
- burn L55.9
- -collapse T67.1
- cramps T67.2
- -excessive T67.9, T67.8
- exhaustion T67.5, T67.3
- stroke T67.01 , exertion T67.02
- syncope T67.1
- heat due to salt depletion T67.4
- heat due to water depletion T67.3Collapse R55- heat T67.1

Appendix B: Disease Groupers:

Obesity PL (List (if any) Problem list ICD-10 codes defined by EDG grouper 1779921)

Cardiovascular PL (List of Problem list ICD-10 codes defined by EDG grouper 1711304)

Respiratory PL (List of Problem list ICD-10 codes defined by EDG grouper 1779908)

Diabetes PL (List of Problem list ICD-10 codes defined by EDG grouper 1139905)

Substance Use PL (List of Problem list ICD-10 codes defined by EDG grouper 1751027)

Contact burn EI (List of ED Impression ICD-10 codes defined by EDG grouper 1138016)

Rhabdomyolysis EI (List of ED Impression ICD-10 codes defined by EDG grouper 117355)

Altered mental status EI (List of ED Impression ICD-10 codes defined by EDG grouper 116565)

Appendix C: Medication Classification Schema

Anticholinergic:

TIOTROPIUM BROMIDE, IPRATROPIUM-ALBUTEROL, ATROVENT, HYOSCYAMINE, SOLIFENACIN, OXYBUTYNIN, CYCLOPENTOLATE, ATROPINE, GLYCOPYRRONIUM, GLYCOPYRROLATE, SCOPOLAMINE, TRIHEXYPHENIDYL, BENZTROPINE, COGENTIN, DICYCLOMINE, BENTYL, SPIRIVA RESPIMAT, LOPERAMIDE

Antihistamine:

HYDROXYZINE, PROMETHAZINE, LORATADINE, CETIRIZINE, DIPHENHYDRAMINE, MECLIZINE

Antipsychotic:

QUETIAPINE, GEODON, RISPERIDONE, HALOPERIDOL, PALIPERIDONE PALMITATE, ARIPIPRAZOLE, FLUPHENAZINE, FLUPHENAZINE DECANOATE, ZYPREXA, ARIPIPRAZOLE

Benzodiazepines:

Lorazepam, CLONAZEPAM, TEMAZEPAM, ALPRAZOLAM

Beta Blockers:

PROPRANOLOL, CARVEDILOL, ATENOLOL, METOPROLOL TARTRATE,

DORZOLAMIDE-TIMOLOL

CCB:

AMLODIPINE

Diuretics:

HYDROCHLOROTHIAZIDE, METOLAZONE

Laxatives:

POLYETHYLENE GLYCOL, BISACODYL, LACTULOSE, SENNOSIDES, DOCUSATE SODIUM

Lithium

SSRI:

SERTRALINE, FLUOXETINE, PAROXETINE

TCA:

AMITRIPTYLINE
